# Food Intake of Macro and Trace Elements from Different Fresh Vegetables Taken from Timisoara Market, Romania—Chemometric Analysis of the Results

**DOI:** 10.3390/foods12040749

**Published:** 2023-02-08

**Authors:** Gabriel Heghedűș-Mîndru, Petru Negrea, Teodor Ioan Trașcă, Ducu Sandu Ștef, Ileana Cocan, Ramona Cristina Heghedűș-Mîndru

**Affiliations:** 1 Faculty of Food Engineering, University of Life Science “King Mihai I” from Timisoara, 300645 Timisoara, Romania; 2 Faculty of Industrial Chemistry and Environmental Engineering, Polytechnic University of Timisoara, Piata Victoriei, RO, 300006 Timisoara, Romania

**Keywords:** vegetables, macro elements, trace elements, principal components analyzed, assessment of the risk to human health

## Abstract

Vegetable consumption is recommended and encouraged by all nutritionists and doctors across the planet. However, in addition to minerals which are beneficial to the body, certain minerals with a negative influence on human health can sneak in. It is very important that in the case of some minerals their content in vegetables is known, so that the recommended limits are not exceeded. The purpose of this study was to evaluate the macro elements (Na, K, Ca, Mg) and trace elements (Cu, Mn, Fe, Cd, Pb, Zn, Co) in 24 samples of vegetables from four botanical families (*Solanaceae*, Brassicaceae, Apiaceae and Amaryllidaceae), purchased from the market in Timișoara, Romania, both imported products as well as local products. The atomic-absorption-spectrometry technique (FAAS) was used to evaluate the macro elements and trace elements. The values obtained for the macro elements and trace elements were used as input data for the analysis of multivariate data, the principal component analysis (PCA) in which the vegetable samples were grouped according to their contribution of certain mineral elements, as well as according to some of the botanical families to which they belong. At the same time, based on the values obtained for trace elements, an assessment of the risk to human health in terms of consumption of the vegetables studied was carried out. The risk assessment for human health was determined on the basis of the estimated daily dose (EDI), the values of the target hazard coefficient (THQ), the values of the total target hazard coefficient (TTHQ) and the carcinogenic risk (CR). Following the determination of THQ, the values obtained followed the order THQ_With_ > THQ_Cd_ > THQ_Pb_ > THQ_Co_ > THQ_Mn_ > THQ_Zn_ > THQ_Fe_. The results on the content of macro elements and trace elements, as well as the assessment of the risk to human health when consuming the assessed vegetables, were within the limits of European Union (EU) and World Health Organization and Food and Agriculture Organization (WHO/FAO)legislation.

## 1. Introduction

Vegetables are an important source of mineral elements that are essential for the proper development of the human body [1]. Research has shown that the intake of vegetables decreases the risk of developing various types of cancer, cardiovascular disease and mortality of any cause [2]. As indicated in the consumer nutrition guide from Health Canada, United States Department of Agriculture (USDA) and Health and Human Services (HHS), the UK’s National Health Service and other agencies, a diet rich in vegetables, fruits and whole grains, which are nutrient-rich foods, will still provide all the nutrients we need for good nutrition [3]. The World Health Organization and Food and Agriculture Organization (WHO/FAO) recommends eating a minimum of 400 g of vegetables and fruits per day (apart from potato and other starched tubers) to prevent some chronic diseases (heart disease, cancer, diabetes and obesity) [4,5].

Even though nutrient deficiency is one of the major health concerns in both developed and developing countries, exceeding certain limits should not be overlooked [6]. In recent years, environmental contamination by heavy metals has been a global concern, due to their persistence and mobility between the biotic and abiotic spheres. “Food intake of plant-derived foods is a major fraction of human exposure that can endanger health”. [7] Contamination of food with heavy metals is influenced by the following factors: the composition of the soil, the genotype of the plant, the environment, and the type of fertilizers and pesticides used [7,8,9]. The management of fertilizers and manure is relevant for the production of food, but simultaneously causes effects on the environment, due to the negative effects on air, soil and water quality. The impact on soil quality is mainly due to the addition of heavy metals, which can have an impact on soil biodiversity and, in the case of Cd, on food quality [10]. Research carried out in Romania as well as in other countries in the world has shown that the highest concentrations of trace elements were obtained in the case of samples of vegetables and fruits grown in soils near mining operations as well as industrial areas [11,12,13].

The main minerals essential for humans are calcium (Ca), phosphorus (P), potassium (K), sodium (Na) and magnesium (Mg), while iron (Fe), copper (Cu), zinc (Zn), manganese (Mn), iodine (I) and selenium (Se) are trace elements [14]. Essential trace elements are important for the biochemical and physiological functions of the human body [1]. Ca is a major contributor in the prevention of cancer, as well as bone health. The latest research has determined that high Ca levels are a risk factor for cardiovascular disease [15]. Another important element from a physiological point of view is Mg, which plays a key role in muscle contraction, gland secretion and nerve transmission. The percentage of Mg existing in the body is 70% in the skeleton, with the rest in the cells. It plays a protective role against cardiovascular disease by increasing endothelium-dependent vasodilation, improving lipid metabolism, reducing systemic inflammation and inhibiting platelet aggregation [15]. Na and K have an effect on the regulation of blood-pressure levels [16,17]. Na and K play a fundamental role in the distribution of fluids inside and outside cells. Exposure to high levels of Na and K is maintained by the specific permeability of cell membranes and by the activity of transmembrane transporters such as Na/K-ATPase. K is an essential mineral and has a primary role in physiological mechanisms, including the transmission of electrical activity in muscle cells and nerve fibers [17,18]. Zn is an essential element for human metabolism that functions as an enzymatic cofactor, contributes to the structure of proteins and regulates the expression of genes [6]. Fe is a vital component of proteins, hemoglobin and myoglobin, and is responsible for transporting oxygen, cellular metabolism, glucose metabolism and vascular functions. Fe deficiency in humans leads to a number of health problems, including the weakening of the immune system and the inhibition of hemoglobin, leading to anemia, insomnia and other health disorders [19]. Copper (Cu) is an essential trace element in both humans and animals. The human body contains approximately 100 mg Cu. Diets with Cu deficiency have serious lifelong consequences. Both in childhood and throughout life, these diets affect the development of the cardiovascular system, the appearance of bone malformations and neurological anomalies, as well as immunological ones. Although it is an essential micronutrient for humans, it can become toxic in the case of a high level. Exposure to high levels of Cu results in the development of redox Fenton-type reactions, leading to damage to oxidative cells and their death [20]. Manganese (Mn) works as a cofactor for a variety of enzymes, including arginase, glutamine synthase, pyruvate carboxylase and Mn superoxide dismutase (MnSOD). However, compared to the shortcomings of other essential micronutrients, such as Fe and Zn, which can develop major health problems, Mn deficiency in humans is rare. However, Mn poisoning can be more common in overexposure to this metal, resulting in liver cirrhosis, polycythemia, dystonia and Parkinson’s-like symptoms [21,22]. Even in small quantities, cadmium (Cd) is a very dangerous element for human health, especially when it is accumulated in kidneys, lungs and liver. Cd contamination of vegetables is caused by frequent use of phosphate fertilizers in agriculture. According to the International Agency for Research on Cancer, Cd belongs to “Group 1” and is considered a precursor to human cancer, even through a low exposure in food [23]. Co deficiency has the effect of developing anemia in pregnant women, as Co stimulates the production of red blood cells, fights anemia, severe fatigue, shortness of breath and hypothyroidism, all of which result from a lack of Co. However, it can cause angina, asthma, cardiomyopathy, polycythemia and dermatitis [24]. Pb has been identified as a toxic metal at high concentrations, and widespread use of Pb results in widespread environmental contamination and global health problems. Having a cumulative toxic effect, Pb can influence the neurological system and kidneys and blood circulation, especially in children, infants and fetuses. Pb is distributed in the brain, liver, kidneys and bones. Over time, Pb accumulates in the teeth and bones, reflecting cumulative human exposure. Pb can also affect the brain and intellectual development in children, inducing apoptosis in the organs’ tissues [25,26,27,28].

Although there are a number of studies in which the content of macro elements and trace elements of different varieties of vegetables has been evaluated, the information published on this topic is limited. Most of the research carried out at international, and especially national level, evaluates the trace-element content of products of plant origin from contaminated areas. Even if some evaluations show trace elements within the limits of international and national legislation, the increased levels for elements such as Pb and Cd must be constantly monitored, compared with data available in the literature, and carefully treated.

The purpose of this study was to determine the levels of macro elements (Na, K, Ca, Mg) and trace elements (Cu, Mn, Fe, Cd, Pb, Zn, Co) in vegetables taken from supermarkets in Timisoara (imported vegetables) and vegetables taken from local producers, from agro-food markets in Timisoara (domestic production), using flame atomic absorption spectrometry (FAAS). The vegetables selected for the study were the following: *Solanacee* (tomato—*L. esculentum*, bell pepper—*C. annuum*, eggplant—*S. melongena*, potato—*S. tuberosum*); *Brassicaceae* (cauliflower—*Brassica oleracea*, white cabbage—*Brassica oleracea* var. *capitata*, kohlrabie—*Brassica oleracea* var. *gongyloides*.); *Apiaceae* (parsley—*P. crispum*, carrot—*Daucus carota* subsp. *Sativus*, celery—*A. graveolens*); and *Amaryllidaceae* (garlic—*A. sativum*, onion—*A. cepa*). A multivariate data analysis, principal component analysis (PCA), was used to determine the association between elements in vegetable samples. The risk assessment for human health was determined on the basis of the estimated daily dose (EDI), the values of the target hazard quotient (THQ), the values of the total target hazard coefficient (TTHQ) and the carcinogenic risk (CR).

## 2. Materials and Methods

### 2.1. Reagents and Materials

All reagents used to determine macro elements and trace elements in the vegetable samples were of extremely pure quality, purchased from Merck (Darmstadt, Germany): nitric acid (65% HNO_3_) and hydrochloric acid (37% HCl). Acetylene was purchased from Linde, Romania, purity 99.6%; the water used was deionized at a resistivity of 18.2 MΩ·cm^−1^ in a Milli-Q^®^ EQ 7008/7016 Ultrapure and pure-water purification system (Merck).

### 2.2. Sample Collection and Preparation Process

The selection of vegetable families for evaluation was made according to the degree of their consumption in the western region of Romania. The selected vegetable families were the following: *Solanacee* (tomato—*L. esculentum*, bell pepper—*C. annuum*, eggplant—*S. melongena*, potato—*S. tuberosum*); *Brassicaceae* (cauliflower—*Brassica oleracea*, white cabbage—*Brassica oleracea* var. *capitata*, kohlrabi—*Brassica oleracea* var. *gongyloides*.); Apiaceae (parsley—*P. crispum*, carrot—*Daucus carota* subsp. *Sativus*, celery—*A. graveolens*); *Amaryllidaceae* (garlic—*A. sativum*, onion—*A. cepa*). The same vegetable families were purchased from local producers in Timis County, Timisoara agro-food market (domestic product—d.p.), and from supermarkets in Timisoara (imported product—i.p.).

After purchase, the vegetable samples were subjected to a conditioning operation to remove impurities (soil or remnants of vegetation, etc.), followed by washing with high-quality reagent water (resistivity 18.2 MΩ·cm^−1^). Vegetable samples were minced using a polypropylene manual grater, then mixed to homogenize the composition and stored in porcelain containers until the analysis. For the determination of the dry matter, 100 g of each sample of vegetables prepared as described above were weighed to the nearest ±0.001 g in porcelain dishes which had been previously cleaned, and dried to a constant weight. The containers with the vegetable samples were kept in a drying chamber (BINDER GmbH, Tuttlingen, Germany,) at 105 °C for 6 h. After this time, the containers with the vegetable samples were removed from the oven and cooled in a desiccator with a drying agent. This step was repeated until the difference between the last two successive weights did not exceed ± 0.001 g [24]. Moisture and dry-matter content were calculated according to Formulas (1) and (2) [29]:

(1) $$\mathrm{Humidity}=\frac{{(G}_{1}-G_{2})}{{(G}_{1}-G_{3})}\cdot 100\;(\%)$$

Dry matter = 100 − Humidity (%)
(2)

where

G_1_ is the weight of the porcelain container and sample before drying;

G_2_ is the weight of the porcelain container and sample after drying;

G_3_ is the weight of the porcelain container.

### 2.3. Dry and Wet Mineralization

*Dry mineralization.* For each plant product, 2 g of dry matter obtained after drying was taken and placed in a porcelain crucible. The porcelain crucibles with the vegetable samples were placed in a calcination kiln where the temperature was gradually increased, in the first step from 200 to 250 °C, and in the second step up to 550 °C for a period of 8 h, until the white ash was obtained.

*Wet mineralization.* After cooling the crucibles with ashes, an amount of 10 mL HCl and 5 mL HNO_3_ was added. It was evaporated until an almost dry sample was obtained.

The solution obtained after mineralization was transferred quantitatively into a volumetric flask of 100 mL, and the flask was filled up to the mark [29].

### 2.4. Macro Element and Trace-Element Determination

The determination of the content of macro elements and trace elements was made using a flame-type atomic absorption spectrometer—Varian 280 FS SpectrAA—, and air-acetylene (FAAS) equipment equipped with lamps for each element. Measurements were carried out at 589.0 nm for Na, 766.5 nm for K, 422.7 nm for Ca, 202.6 nm for Mg, 324.8 nm for Cu, 279.5 nm for Mn, 248.3 nm for Fe, 228.8 nm for Cd, 217.0 nm for Pb, 213.9 nm for Zn and 240.7 nm for Co. Calibration curves were created using five concentration levels of dilutions of standard solutions: for Na, Ca and Mg—5, 10, 15, 20, 25 mg·L^−1^, for K, Pb and Co—2, 4, 6, 8, 10 mg·L^−1^, for Fe—3, 6, 9, 12, 15 mg·L^−1^, for Cu and Mn—1, 2, 3, 4, 5 mg·L^−1^, and for Zn and Cd—0.5, 1, 1.5, 2, 2.5 mg·L^−1^. The analyses of the samples were carried out in triplicate. The results obtained are presented in g·Kg^−1^ dry weight for the macro elements and μg·g^−1^ dry weight for the trace elements.

### 2.5. Assessment of the Risk to Human Health

Due to the lack of clear recommendations in the Romanian legislation for the amount of fresh vegetables consumed every day, but also due to the lack of statistics regarding this consumption, we considered 200 g vegetables as representing half the amount of fruits and vegetables recommended by the WHO/FAO [4,5].

The potential risk to human health due to vegetable consumption was assessed using the target hazard quotient (THQ), and was calculated using Equation (3) [7,11,30,31]

(3) $$\mathrm{THQ}=\frac{\mathrm{EDI}}{\mathrm{RfD}}$$

where EDI is the estimated daily dose in μg·Kg^−1^·day^−1^ and RfD is the reference dose; the RfD is: Co—20 μg·Kg^−1^·day^−1^, Cd—1 μg·Kg^−1^·day^−1^, Pb—3.57 μg·Kg^−1^·day^−1^, Zn—300 μg·Kg^−1^·day^−1^, Mn—140 μg·Kg^−1^·day^−1^, Fe—45,000 μg·Kg^−1^·day^−1^ and Cu—40 μg·Kg^−1^·day^−1^ [32,33,34,35,36,37]

The estimated daily intake of trace elements (EDI) is calculated using Equation (4)

(4) $$\mathrm{EDI}=\frac{C\cdot \mathrm{IR}\cdot \mathrm{EF}\cdot \mathrm{ED}}{\mathrm{BW}\cdot \mathrm{AT}}$$

where C is the metal concentration in the sample in μg·Kg^−1^, IR is the ingestion rate (vegetables/day as half of the recommended amount by WHO/FAO selected in mg/day), EF is the frequency of exposure (365 days per year), ED is the duration of exposure (70 years), BW is body weight (70 Kg) and AT is the average exposure time (EF × ED) [5,7,11,30,31].

If the THQ is below the value of 1, the risk to human health is low, even for sensitive people, and if the THQ is equal to or greater than 1, risks to the health of consumers may arise.

TTHQ is the sum of THQ of all trace elements, and was intended to assess the cumulative effect and the potential health risk in the case of exposure to a mixture of trace elements.

TTHQ located above a value of 1 indicates a significant health problem [33].

TTHQ is calculated using Equation (5) [7,11,30,31].

(5) $$\mathrm{TTHQ}=\mathrm{TQH}\left(\mathrm{Cu}\right)+\mathrm{TQH}\left(\mathrm{Mn}\right)+\mathrm{TQH}\left(\mathrm{Fe}\right)+\mathrm{TQH}\left(\mathrm{Cd}\right)+\mathrm{TQH}\left(\mathrm{Pb}\right)+\mathrm{TQH}\left(\mathrm{Zn}\right)+\mathrm{TQH}\left(\mathrm{Co}\right)$$

Carcinogenic risk (CR) is defined as a person’s likelihood of developing cancer over the course of his life due to exposure to metals. In the case of this evaluation, only the elements Cd and Pb are considered to be precursors of cancer.

The assessment of carcinogenic risk can be carried out using Equation (6)

(6) CR=SF·EDI

where EDI is the estimated daily intake of metal ingested through vegetables, and SF is the carcinogenic slope factor. SF values were 14 μg Kg^−1^ day^−1^ (Cd) and 8.5 μg Kg^−1^ day^−1^ Pb [7,11,30,31,38].

### 2.6. Statistical Approach

Principal component analysis (PCA): in cases where the dataset contains a large number of dependent variables, it is recommended that the dataset be reduced to smaller segments, thus providing a clearer and easier-to-interpret result.

The analysis of the principal components (PCA) is an ideal tool for such problems, as a dataset can be described by the main components, depending on the degree of variation within the data; this produces a reduction in the size of the data, and allows for the visualization of the basic structure of the data, as it indicates the experimental relationships between the data and the samples.

Statistical analysis and graphical representations were conducted using the Origin Pro 2020 package software (Stat-Ease Inc., Minneapolis, MN, USA) [29,39].

Statistical differences between sample parameters (V1–V24) were assessed, using a one-way ANOVA followed by a two-sample *t*-test with equal variance. Results of statistical analyses between samples (V1–V24) were reported in tables in the same column with different exponents where significant differences were identified (*p* < 0.05). Data presented in the same column with the same exponents or letters showed no significant differences (*p* > 0.05). The statistical tool used for data processing was Microsoft Excel 365 (version 2208, Redmond, WA, USA).

## 3. Results and Discussion

### 3.1. Concentration of Macro and Trace Elements

In the first stage, 12 species of vegetables from four botanical families were selected. The analyses performed to determine the water content showed values in the range (61.82–95.31%) with an average of 88.02% in the case of the 24 vegetable samples. The results obtained in terms of water content, macro elements and trace elements for the 24 samples of vegetables are presented in Table 1 and Table 2.

The most abundant elements in the evaluated vegetables were Na, K, Ca and Mg. Na was determined for this work with values ranging from 0.49 to 11.40 g·Kg^−1^ d.w., with an average of 5.94 Kg^−1^ d.w., the lowest value being recorded in the bell pepper i.p. sample and the highest value in the carrot i.p. These values are comparable to those obtained in the United Kingdom for several onion samples in the range of 0.03–0.14 g·Kg^−1^ d.w. [42]. In the Pakistan Punjab area, several samples of bell pepper and onion indicated values for Na in the range of 5.8–12.8 g·Kg^−1^ d.w. [43]. In Poland, several potato cultivars indicated values in the range of 0.66–1.61 g·Kg^−1^ d.w. [44].

K was determined in this paper with values ranging from 16.70 to 43.70 g·Kg^−1^ d.w., with an average of 30.2 g·Kg^−1^ d.w., the lowest value being recorded in the onion d.p. sample, and the highest value in the potato i.p. sample. These values are comparable to those obtained in the Pakistan Punjab area, where samples of tomato, bell pepper and onion indicated values for K in the range of 4.2–33.4 g·Kg^−1^ d.w. [43], and in Poland, where several potato cultivars indicated values in the range of 11.59–24.34 g·Kg^−1^ d.w. [44]. In Latvia, onion samples indicated values in the range of 9.85–28.41 g·Kg^−1^ d.w, with carrot in the range of 2.31–3.91 g·Kg^−1^ d.w. [45].

Ca was determined in this work with values between 0.016 and 0.90 g·Kg^−1^ d.w., with an average of 0.45 g·Kg^−1^ d.w., the lowest value being recorded in the parsley d.p. sample and the highest value in the eggplant sample i.p. These values are comparable to those obtained in the United Kingdom for several onion samples in the range of 0.2 to 0.6 g·Kg^−1^ d.w. [46]. In Poland, several potato cultivars indicated values in the range of 11.59–24.34 g·Kg^−1^ d.w. [44]. In Latvia, onion samples indicated values in the range of 9.85–28.41 g·Kg^−1^ d.w., with carrot in the range of 2.31–3.91 g·Kg^−1^ d.w. [45].

Mg was determined in this paper with values between 0.32 and 1.66 g·Kg^−1^ d.w., with an average of 0.99 g·Kg^−1^ d.w., the lowest value being recorded in the tomato i.p. sample and the highest value in the parsley i.p. sample. These values are comparable to those obtained in Poland, where samples of garlic indicated values for Mg of 0.23 g·Kg^−1^ d.w., and onion samples indicated 0,06 g·Kg^−1^ d.w. [47]; also in Poland, several potato cultivars indicated values in the range of 1.46–1.84 g·Kg^−1^ d.w. [44], while in the United Kingdom, onion samples were in the range of 0.04–0.1 g·Kg^−1^ d.w. [42].

Following the determination of macro elements, the obtained values followed the order K > Na > Mg > Ca.

Cu was determined in this paper with values between 0.5 and 76.50 μg·g^−1^ d.w., with an average of 38.5 μg·g^−1^ d.w., the lowest value being recorded in the sample of carrot i.p. and the highest value in the sample of eggplant i.p. These values are comparable to those obtained in France, in supermarkets in the city of La Rochelle, for samples of green pepper—51.34 μg·g^−1^ d.w., carrot—30.38 μg·g^−1^ d.w., eggplant—44.36 μg·g^−1^ d.w., cabbage—49.55 μg·g^−1^ d.w., potato—25.23 μg·g^−1^ d.w., tomato—104.68 μg·g^−1^ d.w. and onion—70.71 μg·g^−1^ d.w. [48]; in Italy, in the city of Bologna, for several tomato cultivars the values recorded for Cu were located in the range of 11.50–13.10 μg·g^−1^ d.w. [49], and in Finland, for potato samples—6 μg·g^−1^ d.w., carrot—5 μg·g^−1^ d.w. and celery root—12 μg·g^−1^ d.w. [50].

Manganese (Mn) was determined in this work with values ranging from 8 to 29.5 μg·g^−1^ d.w., with an average of 18.7 5 μg·g^−1^ d.w., the lowest value being recorded in the kohlrabie sample i.p. and the highest value in the sample of parsley i.p. These values are comparable to those obtained in Finland for potato samples—7 μg·g^−1^ d.w., carrot—27 μg·g^−1^ d.w. and celery root—15 μg·g^−1^ d.w. [50], in India, in the Pradesh area, for tomato samples—1.6 μg·g^−1^ d.w. [48,51], in northwestern Botswana, two supermarkets in Maun, tomato samples—19.1 μg·g^−1^ d.w., onion samples—19.8 μg·g^−1^ d.w., cabbage—38.6 μg·g^−1^ d.w. and potato—17.2. μg·g^−1^ d.w. [52].

Fe was determined in this paper with values ranging from 18.5 to 110 μg·g^−1^ d.w. with an average of 64.25 μg·g^−1^ d.w., the lowest value being recorded in the onion sample d.p. and the highest value in the cauliflower sample d.p. These values are comparable to those obtained in Iran, where Isfahan samples of bell pepper indicated an Fe content in the range of 45–50 μg·g^−1^ [53], in Finland, for potato samples—34 μg·g^−1^ d.w., carrot—30 μg·g^−1^ d.w., celery root—55 μg·g^−1^ d.w., cauliflower—59 μg·g^−1^ d.w., white cabbage, 53 μg·g^−1^ d.w., onion—28 μg·g^−1^ d.w., sweet pepper—4.1 μg·g^−1^ d.w. and tomato—29 μg·g^−1^ d.w. [50], and Upper Egypt, for tomato samples—19.69 μg·g^−1^ d.w. [54].

Cadmium (Cd) was determined in this paper with values ranging from 0.1 to 0.5 μg·g^−1^ d.w., with an average of 0.3 μg·g^−1^ d.w., the lowest value being recorded in tomato i.p. and bell pepper i.p. samples and the highest value in the bell pepper sample d.p. These values are comparable to those obtained in Denmark, Copenhagen, for several carrot samples with values in the range of 0.09–0.206 μg·g^−1^ d.w. and several samples of potato with values in the range of 0.032–0.088 μg·g^−1^ d.w. [55]; in Macedonia, in the Skopje Usje region, for carrot—1027 μg·g^−1^ d.w., parsley—0.053 μg·g^−1^ d.w., and also in Skopje in the Jurumleri region, for carrot—0.029 μg·g^−1^ d.w., and cauliflower—0.013 μg·g^−1^ d.w. [56], and Spain, for more tomato samples—0.05 μg·g^−1^ d.w., and onion—12 μg·g^−1^ d.w. [57].

Pb was determined in this paper with values ranging from 0.2 to 1 μg·g^−1^ d.w., with an average of 6 μg·g^−1^ d.w., the lowest value being recorded in the cauliflower sample i.p. and the highest value in the cauliflower sample d.p. These values are comparable to those obtained in Spain for several samples of tomato—0.04 μg·g^−1^ d.w., and onion—0.037 μg·g^−1^ d.w. [57], in Macedonia, in the Skopje, Usje region, for parsley—0.031 μg·g^−1^ d.w., and also in the Skopje region, in the city of Jurumleri, for onion—0.078 μg·g^−1^ d.w., and cauliflower—0.023 μg·g^−1^ d.w. [56], and in Serbia, in the Vojvodina Province, for potato—1.13 μg·g^−1^ d.w. [58].

Zn was determined in this paper with values ranging from 0.45 to 28.5 μg·g^−1^ d.w. with an average of 14.47 μg·g^−1^ d.w., the lowest value being recorded in the parsley sample i.p. and the highest value in the cauliflower sample i.p. These values are comparable to those obtained in Egypt, Alexandria, for tomato—7.69 μg·g^−1^ d.w., carrot—8.03 μg·g^−1^ d.w., eggplant—11.5 μg·g^−1^ d.w., garlic—14.9 μg·g^−1^ d.w., onion—11.4 μg·g^−1^ d.w., and potato—7.16 μg·g^−1^ d.w. [59], in Iran for tomato—10 μg·g^−1^ d.w. and bell pepper—48 μg·g^−1^ d.w., [53]; in Poland, samples of garlic indicated values for Zn of 12,2 μg·g^−1^ d.w., and onion samples indicated 4,33 μg·g^−1^ d.w.

Co was determined in this paper with values ranging from 0.5 to 3.55 μg·g^−1^ d.w., with an average of 14.47 μg·g^−1^ d.w., the lowest value being recorded in the cauliflower sample i.p. and the highest value in the white cabbage sample i.p. These values are comparable to those obtained in Finland, for potato samples– 0,08 μg·g^−1^ d.w., carrot—0,04 μg·g^−1^ d.w., celery root—0.03 μg·g^−1^ d.w., cauliflower—0.11 μg·g^−1^ d.w., white cabbage, 0.06 μg·g^−1^ d.w., onion—0.04 μg·g^−1^ d.w., sweet pepper—0.03 μg·g^−1^ d.w. and tomato—0.03 μg·g^−1^ d.w. [50], and in Canada, in the city of Brandon, for carrot samples—0.2 μg·g^−1^ d.w. [60].

Following the determination of trace elements, the obtained values followed the order Fe > Cu > Mn > Zn > Co > Pb > Cd.

The resulting differences can be attributed to the conditions of cultivation, soil composition, fertilizing methods, and the quality of water used for irrigation.

### 3.2. Chemometric Analysis

**Figure 1:**
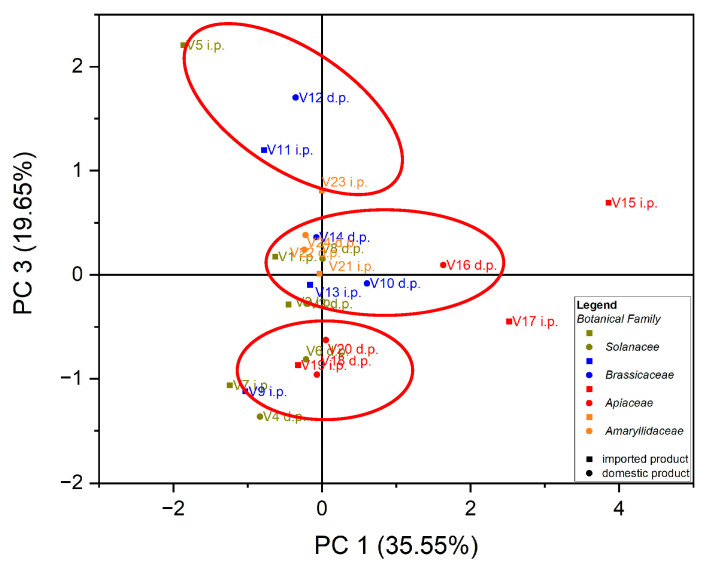
Chart of scores for PC3 vs. PC1 for PCA analysis, using as input data the content of macro elements for the analyzed vegetable samples.

**Figure 2:**
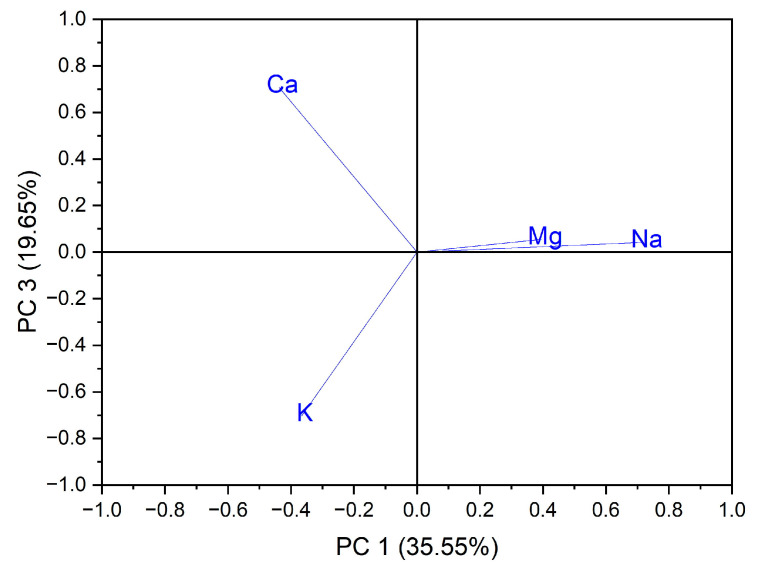
Chart of records of PC3 vs. PC1 for PCA analysis, using as input data the content of macro elements for the analyzed vegetable samples.

Principal component analysis (PCA) is the most common technique used to obtain a basic perspective of the data structure and especially to determine the parameters that have the greatest influence in the classification of samples, as well as their differentiation. In this work, the analysis (PCA) was carried out in two directions: first, for the classification of samples according to the origin of the botanical family to which they belong, and second for the classification of samples according to the evaluated macro elements and trace elements. Using as input data the values of the macro elements (Na, K, Ca and Mg) in the case of the 24 vegetables, the variance in the data was explained by the first three main components, at the rate of 87.97%, as follows: PC1 = 35.55%, PC2 = 32.77% and PC3 = 19.65%. A grouping of samples could not be obtained according to botanical origin. Three groupings of vegetable samples were obtained according to the macro elements assessed, Figure 1. The formation of the first grouping at the top of the PC3 vs. PC1 score graph is dependent on the Ca content of the evaluated vegetable samples. The second group, formed in the center of the graph of the scores PC3 vs. PC1, is dependent on the Mg and Na content of the evaluated vegetable samples. In addition, the third grouping, formed at the bottom left of the PC3 vs. PC1 score graph, is dependent on the high K content of the evaluated vegetables, Figure 2. In the case of analysis of the main components, PC1 vs. PC2 and PC2 vs. PC3, no significant results were obtained in terms of grouping the samples according to their content of macro elements.

**Figure 3:**
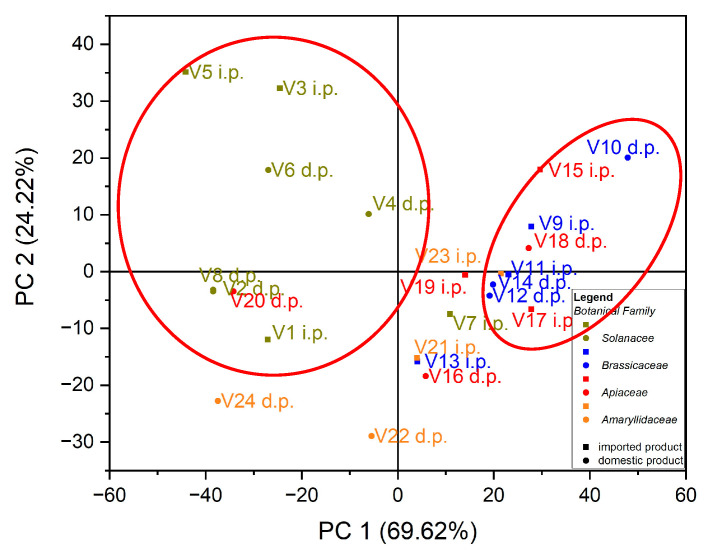
Chart of scores for PC2 vs. PC1 for PCA analysis, using as input data the content of trace elements for the analyzed vegetable samples.

**Figure 4:**
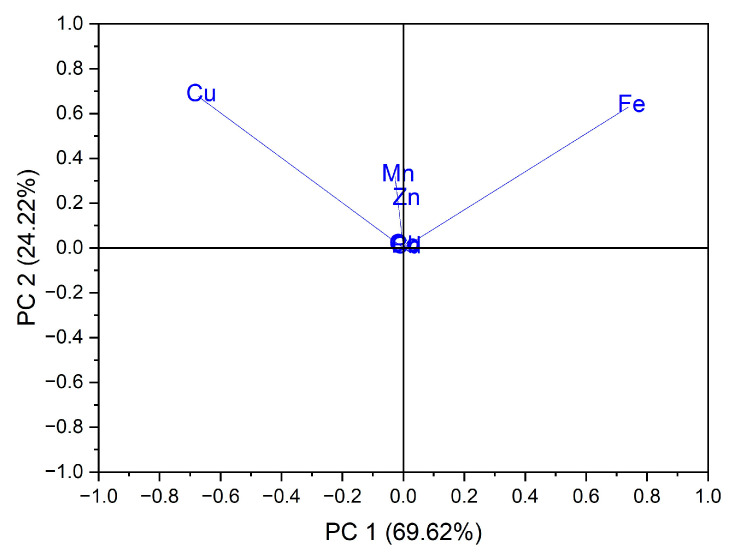
Chart of records of PC2 vs. PC1 for PCA analysis, using as input data the content of trace elements for the analyzed vegetable samples.

Using as input data the values of trace elements (Cu, Mn, Fe, Cd, Pb, Zn and Co) for the 24 vegetables, the variation in the data was explained by the first three main components at a rate of 98.64% as follows: PC1 = 69.62%, PC2 = 24.22% and PC3 = 4.80%. In the case of the analysis of the main components, PC1 vs. PC2, two groups were obtained as follows: one for seven out of eight samples of vegetables from the botanical family Solanaceae, seen in Figure 3; the formation of this group is due to the Cu intake of vegetables from this botanical family, and the second group, for five out of six samples from the botanical family *Brassicaceae*, the formation of this group is due to the Fe intake of vegetables from this botanical family, seen in Figure 4.

**Figure 5:**
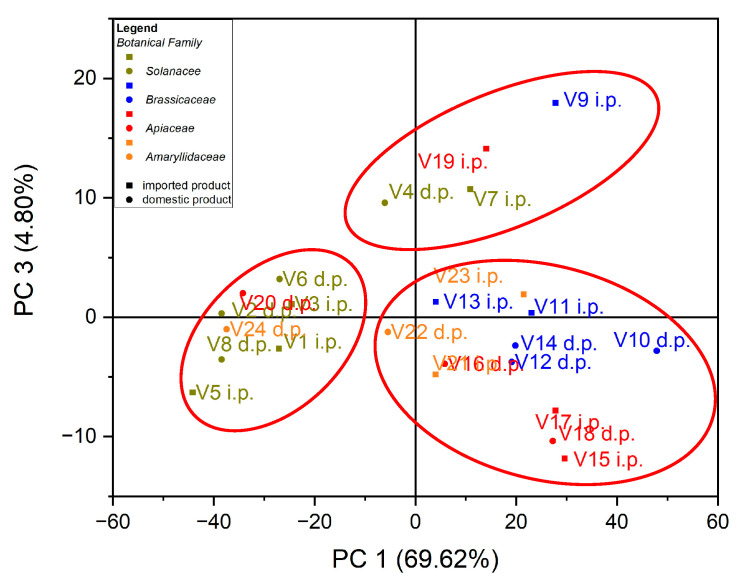
Chart of scores for PC3 vs. PC1 for PCA analysis, using as input data the content of trace elements for the analyzed vegetable samples.

**Figure 6:**
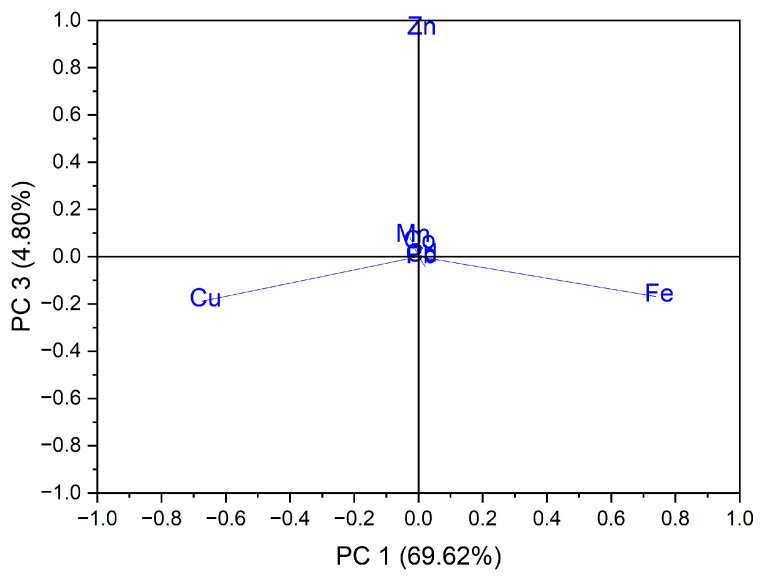
Chart of records of PC3 vs. PC1 for PCA analysis, using as input data the content of trace elements for the analyzed vegetable samples.

In the case of the analysis of the main components, PC3 vs. PC1, three groups of samples indicated in Figure 5 were obtained: for the first group, located at the top of the central part, the formation is due to the Zn content of the evaluated vegetable samples, while for the second group, located on the left side, it is due to the Cu content of the vegetable samples; for the third group, located on the right, the formation is due to the Fe content of the assessed vegetable samples see in Figure 6. In the case of the analysis of the main components, PC2 vs. PC3, no significant results were obtained in terms of grouping of samples according to their content of trace elements.

### 3.3. Assessment of the Risk to Human Health

The trace-element content determined for the 24 vegetables presented in Table 2 was reported for fresh vegetables, for human-health risk assessment. [61]

Table 3 and Table 4 show the results obtained for EDI, THQ, TTHQ and CR for the seven trace elements studied.

**Figure 7:**
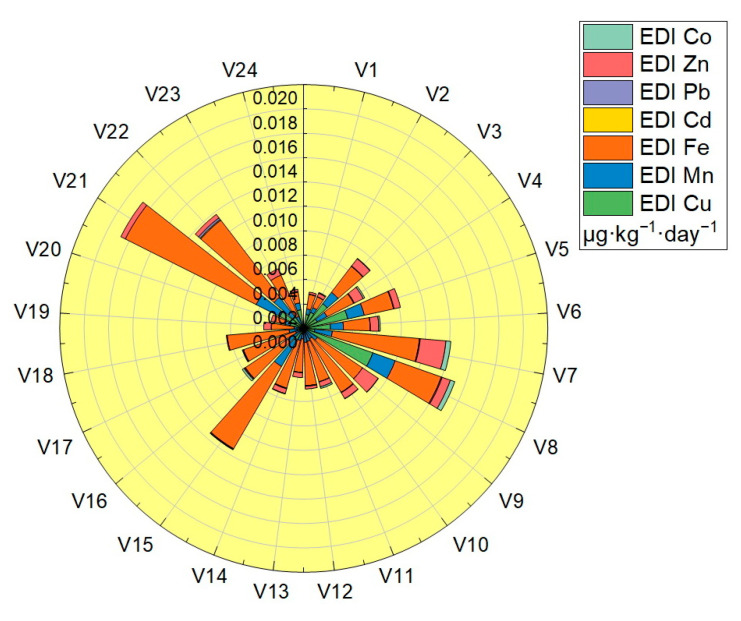
Graphical representation of the estimated daily intake (EDI).

Estimated daily intake (EDI) was calculated in the case of a body weight of 70 Kg for all the assessed vegetable samples: the values obtained are shown in Table 3, and plotted in Figure 7. The EDI values obtained for the analyzed trace elements were located in the following ranges: Cu (2.89 × 10^−5^–6.01 × 10^−3^ μg·Kg^−1^·day^−1^), Mn (4.02 × 10^−4^–2.45 × 10^−3^ μg·Kg^−1^·day^−1^), Fe (8.84 × 10^−4^–7.94 × 10^−3^ μg·Kg^−1^·day^−1^), Cd (3.75 × 10^−6^–8.35 × 10^−5^ μg·Kg^−1^·day^−1^), Pb (1.07 × 10^−5^–1.67 × 10^−4^ μg·Kg^−1^·day^−1^), Zn (3.74 × 10^−5^–2.12 × 10^−3^ μg·Kg^−1^·day^−1^) and Co (2.69 × 10^−5^–1.24 × 10^−4^ μg·Kg^−1^·day ^−1^). The EDI values obtained for each trace element in the vegetable samples analyzed were significantly lower than the reference values (*RfD*): for Cu the highest value obtained was 6.01 × 10^−3^ μg·Kg^−1^·day^−1^, located well below the *RfD_Cu_* value—40 μg·Kg^−1^ ·day^−1^, for Mn the highest value obtained was 2.45 × 10^−3^ μg·Kg^−1^·day^−1^, located well below the *RfD_Mn_* value—140 μg·Kg^−1^ ·day^−1^, for Fe the highest value obtained was 7.94 × 10^−3^ μg·Kg^−1^·day^−1^, located well below the *RfD_Fe_* value—45,000 μg·Kg^−1^ ·day^−1^, for Cd the highest value obtained was 8.35 × 10^−5^ μg·Kg^−1^·day^−1^, located well below the *RfD_Cd_* value—1 μg·Kg^−1^·day^−1^, for Pb the highest value obtained was 1.67 × 10^−4^ μg·Kg^−1^·day^−1^, located well below the *RfD_Pb_* value—3.57 μg·Kg^−1^·day^−1^, for Zn the highest value obtained was 2.12 × 10^−3^ μg·Kg^−1^·day^−1^, located well below the *RfD_Zn_* value—300 μg·Kg^−1^ ·day^−1^ and for Co the highest value obtained was 1.24 × 10^−4^ μg·Kg^−1^·day^−1^, located well below the value of *RfD_Co_*—20 μg·Kg^−1^·day^−1^ [32,33,34,35,36,37].

**Figure 8:**
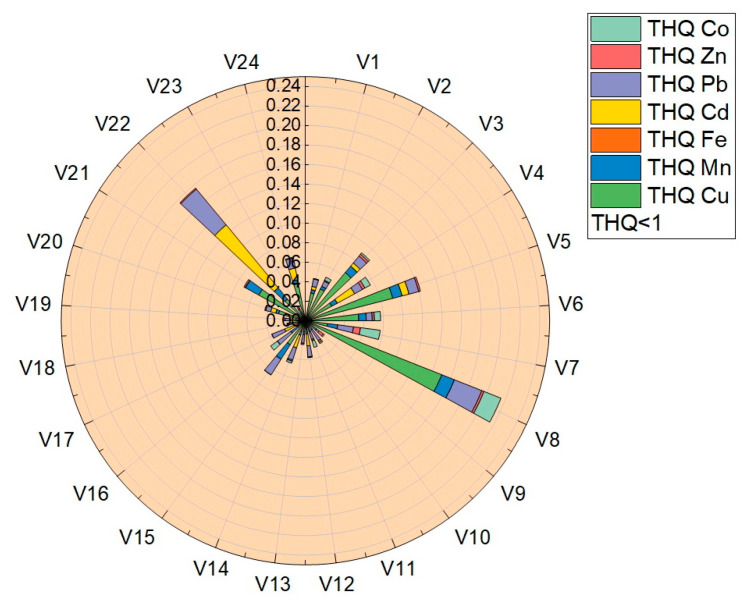
Graphical representation of the target hazard quotient (THQ).

The target-hazard-quotient values (THQ) were calculated for all vegetable samples, the values obtained being presented in Table 4 and plotted in Figure 8. The THQ values obtained for the analyzed trace elements were located in the following ranges: THQ_Cu_ (7.2 × 10^−4^–1.5 × 10^−1^), THQ_Mn_ (2.9 × 10^−3^–1.2 × 10^−2^), THQ_Fe_ (1.97 × 10^−5^–2.67 × 10^−4^), THQ_Cd_ (3.57 × 10^−3^–8.35 × 10^−2^), THQ_Pb_ (3.07 × 10^−3^–4.77 × 10^−2^), THQ_Zn_ (1.25 × 10^−4^–7.07 × 10^−3^) și THQ_Co_ (1.34 × 10^−3^–1.87 × 10^−2^). Following the determination of the THQ, the obtained values followed the order THQ_Cu_ > THQ_Cd_ > THQ_Pb_ > THQ_Co_ > THQ_Mn_ > THQ_Zn_ > THQ_Fe_.

**Figure 9:**
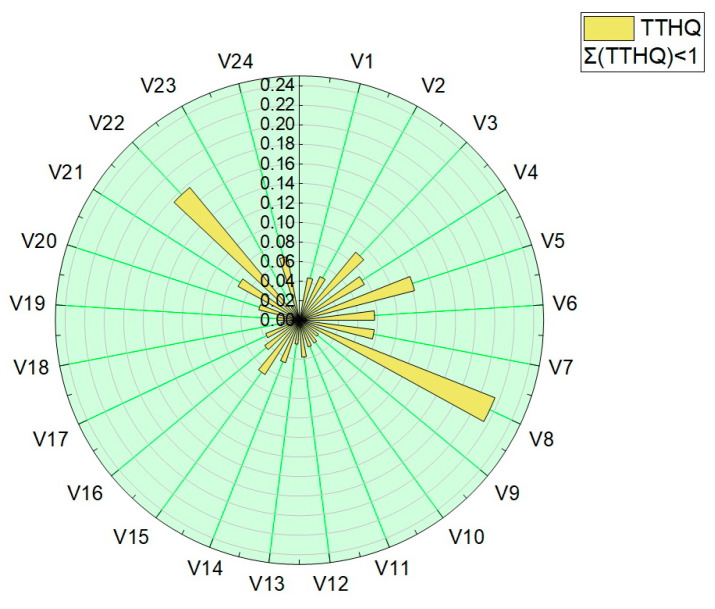
Graphical representation of the sum of the target hazard quotient (TTHQ).

The total target-hazard-quotient (TTHQ) values were calculated for all vegetable samples, and are presented in Table 4 and plotted in Figure 9. The values obtained for the total target hazard quotient (TTQH) were in the range 1.56 × 10^−2^–2.15 × 10^−1^.

All values obtained for the target hazard quotient (TQH) and the total target hazard quotient (TTQH) were below 1, indicating that there are no significant health risks associated with the intake of trace elements or their mixture by eating the assessed vegetables.

**Figure 10:**
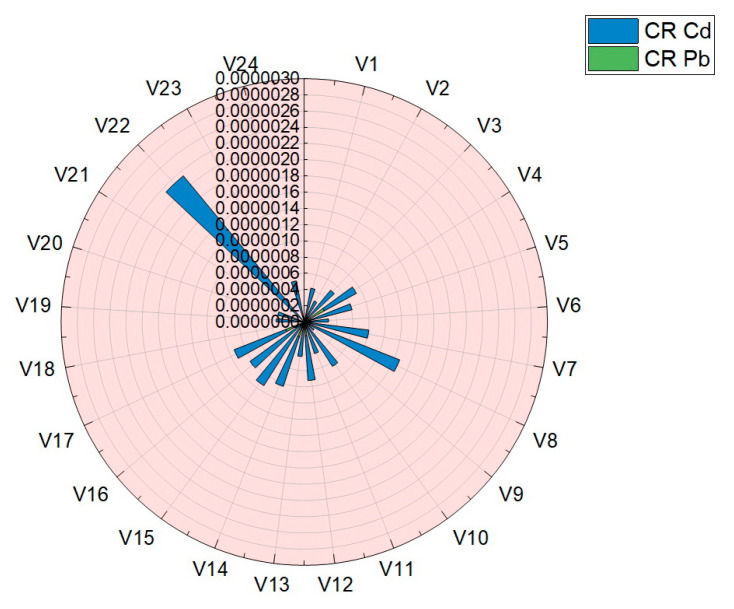
Graphical representation of the results obtained for carcinogenic risk (CR) in the case of Pb and Cd.

Carcinogenic risk (CR) is aimed at assessing an individual’s increased likelihood of developing cancer throughout his life, due to the ingestion of vegetables analyzed in this paper. The results obtained for the assessment of carcinogenic risk (CR) are presented in Table 4 and plotted in Figure 10. The values obtained for the assessment of the carcinogenic risk in the case of consumption of vegetables studied in this work were situated in the following ranges: Cd (4.99 × 10^−8^–2.82 × 10^−7^) and Pb (1.50 × 10^−7^–2.34 × 10^−6^). For the two assessed elements, Cd and Pb, the carcinogenic risk index (CR) was well below the limits imposed by the legislation and norms: 14 μg·Kg^−1^·Day^−1^ Cd and 8.5 μg·Kg^−1^·Day^−1^ Pb [7,11,30,31,38].

## 4. Conclusions

The research carried out in this paper evaluated 24 vegetables from four botanical families (Solanaceae, Brassicaceae, Apiaceae and Amaryllidaceae), taken from local producers (domestic product) and supermarkets (imported product) regarding the content of macro elements (Na, K, Ca and Mg) and trace elements (Cu, Mn, Fe, Cd, Pb, Zn and Co), using the FAAS evaluation technique. The macro elements showed the highest content for the analyzed vegetable samples, being an important source of K, Na, Mg and Ca. Comparison of the present results with those obtained by other researchers were fairly close, especially in case of macro elements.

For most of the samples there were significant differences between i.p. and d.p. for both major-element and trace-element content, with no clear rule influencing these differences.

PCA analysis was carried out in two directions: the first for the classification of samples according to the origin of the botanical family to which they belong, and the second for the classification of samples according to the macro elements and trace elements evaluated. Using as input data the values of the macro elements (Na, K, Ca and Mg) for the 24 vegetables, the results are composed of three groups of vegetables, as follows: the first group is influenced by the content of the samples in Ca, the second group is influenced by the Mg and Na content, and the third group by the content of the vegetable samples in K. A grouping of samples could not be obtained on the basis of botanical origin.

Using as input data the trace-element values (Cu, Mn, Fe, Cd, Pb, Zn and Co) of 24 vegetable samples, two groups were obtained as follows: one for seven out of eight samples of vegetables from the botanical family Solanaceae, the importance of this group being the intake of these vegetables for Cu; the second group comprising five out of six vegetables of the botanical family Brassicaceae, the importance of this group being the intake of these vegetables for Fe.

The values obtained for the trace-element content of 24 vegetable samples taken from local producers (domestic product) and supermarkets (imported product) were used as the input to assess the risk to human health in terms of their consumption. The estimated daily intake (EDI) was calculated in the case of a body weight of 70 Kg for all the assessed vegetable samples. The EDI values obtained for each trace element in the vegetable samples analyzed were significantly lower than the RfD reference values. The target hazard quotient (THQ) and the total target hazard quotient (TTHQ) were calculated for all vegetable samples, the results obtained being well below the limit value of 1, indicating that there are no significant health risks associated with the intake of trace elements or their mixture by eating the vegetables assessed.

The results obtained for the carcinogenic-risk assessment (CR) for the two assessed elements, Cd and Pb, were well below their baselines.

The results obtained in this work for the content in macro elements and trace elements, as well as the assessment of the risk to human health of the 24 samples of vegetables from four botanical families (Solanaceae, Brassicaceae, Apiaceae and Amaryllidaceae), taken from local producers (domestic product) and supermarkets (imported product), were situated within the limits of the EU and WHO/FAO legislation, taking into consideration an amount of daily consumed vegetables of 200 g, representing half the amount of fruits and vegetables recommended by the WHO/FAO.

## Figures and Tables

**Table 1:** The macro elements and moisture content in the 24 vegetable samples of different botanical families (g·Kg^−1^ dry weight, mean ± SD).

Sample Coding	Vegetable Samples Analyzed	Moisture Content (%)	Na	K	Ca	Mg
	Solanaceae					
V1	Tomato (*L. esculentum*) i.p.	93.76	0.68 ± 0.13 ^a,A^	21.75 ± 2.29 ^a,A^	0.16 ± 0.02 ^a,A^	0.32 ± 0.01 ^a,A^
V2	Tomato (*L. esculentum*) d.p.	94.52	2.21 ± 0.54 ^b,B^	28.40 ± 4.17 ^b,B^	0.17 ± 0.02 ^a,A^	0.57 ± 0.09 ^b,c,B^
V3	Bell Pepper (*C. annum*) i.p.	92.52	0.49 ± 0.22 ^c,A^	25.25 ± 0.17 ^c,A^	0.10 ± 0.03 ^b,A^	0.52 ± 0.04 ^b,A^
V4	Bell Pepper (*C. annum*) d.p.	92.94	0.77 ± 0.19 ^a,B^	39.40 ± 2.40 ^d,B^	0.12 ± 0.02 ^b,A^	0.67 ± 0.04 ^c,d,B^
V5	Eggplant (*S. melongena*) i.p.	91.55	1.62 ± 0.37 ^d,A^	27.85 ± 1.23 ^b,A^	0.90 ± 0.07 ^c,A^	0.56 ± 0.05 ^b,A^
V6	Eggplant (*S. melongena*) d.p.	92.50	1.54 ± 0.25 ^d,A^	32.70 ± 1.83 ^e,B^	0.11 ± 0.05 ^b,B^	0.75 ± 0.09 ^d,B^
V7	Potato (*S. tuberosum*) i.p.	79.93	0.99 ± 0.35 ^e,A^	43.70 ± 2.54 ^f,A^	0.31 ± 0.05 ^d,A^	0.76 ± 0.09 ^d,A^
V8	Potato (*S. tuberosum*) d.p.	77.40	0.93 ± 0.40 ^e,A^	18.35 ± 0.24 ^g,B^	0.06 ± 0.006 ^e,B^	0.49 ± 0.05 ^b,B^
	Brassicaceae					
V9	Cauliflower (*B. oleracea*) i.p.	90.60	1.33 ± 0.09 ^f,A^	42.85 ± 2.97 ^f,A^	0.27 ± 0.007 ^d,f,A^	0.76 ± 0.07 ^c,A^
V10	Cauliflower (*B. oleracea*) d.p.	91.90	4.56 ± 0.61 ^g,B^	30.00 ± 1.90 ^h,B^	0.24 ± 0.02 ^f,A^	0.88 ± 0.12 ^e,B^
V11	White cabbage (*B. oleracea* var. *capitata*) i.p.	92.13	0.93 ± 0.23 ^e,A^	29.35 ± 3.00 ^h,A^	0.61 ± 0.06 ^g,A^	1.13 ± 0.16 ^f,A^
V12	White cabbage (*B. oleracea* var. *capitata*) d.p.	91.40	1.84 ± 0.25 ^d,B^	18.15 ± 0.95 ^g,B^	0.50 ± 0.07 ^h,B^	0.72 ± 0.03 ^d,B^
V13	Kohlrabie (*B. oleracea* var. *gongyloides*) i.p.	91.12	0.57 ± 0.26 ^a,c,A^	20.90 ± 2.40 ^a,A^	0.05 ± 0.070 ^e,A^	0.50 ± 0.06 ^b,A^
V14	Kohlrabie (*B. oleracea* var. *gongyloides*) d.p.	90.53	2.76 ± 0.31 ^h,B^	24.45 ± 2.51 ^c,B^	0.26 ± 0.04 ^d,f,B^	0.57 ± 0.04 ^b,c,A^
	Apiaceae					
V15	Parsley (*P. crispum)* i.p.	85.47	8.97 ± 0.76 ^i,A^	15.00 ± 0.77 ^i,A^	0.04 ± 0.007 ^e,A^	1.66 ± 0.27 ^g,A^
V16	Parsley (*P. crispum)* d.p.	86.20	4.59 ± 0.98 ^g,B^	18.95 ± 2.36 ^g,B^	0.016 ± 0.002 ^i,B^	0.94 ± 0.01 ^h,B^
V17	Carrot (*D. carota* subsp. *Sativus*) i.p.	89.89	11.40 ± 2.97 ^j,A^	26.20 ± 3.39 ^b,c,A^	0.027 ± 0.04 ^j,A^	0.48 ± 0.04 ^b,A^
V18	Carrot (*D. carota* subsp. *Sativus*) d.p.	89.66	2.03 ± 0.37 ^b,B^	30.70 ± 1.20 ^h,B^	0.025 ± 0.002 ^j,A^	0.56 ± 0.02 ^b,c,A^
V19	Celery (*A. graveolens*) i.p.	95.21	1.36 ± 0.33 ^f,A^	35.15 ± 7.45 ^j,A^	0.15 ± 0.03 ^b,k,A^	0.85 ± 0.10 ^c,h,A^
V20	Celery (*A. graveolens*) d.p.	95.31	1.29 ± 0.36 ^f,A^	34.60 ± 2.19 ^j,A^	0.19 ± 0.04 ^a,k,A^	1.20 ± 0.21 ^g,B^
	Amaryllidaceae					
V21	Garlic (*A. sativum*) i.p.	61.82	0.80 ± 0.07 ^e,A^	17.85 ± 1.44 ^g,k,A^	0.01 ± 0.003 ^i,A^	0.39 ± 0.01 ^a,A^
V22	Garlic (*A. sativum*) d.p.	63.45	0.67 ± 0.19 ^a,B^	18.30 ± 1.55 ^g,A^	0.09 ± 0.007 ^e,B^	0.40 ± 0.07 ^a,A^
V23	Onion (*A. cepa*) i.p.	91.50	1.24 ± 0.33 ^f,A^	17.80 ± 1.27 ^g,k,A^	0.23 ± 0.02 ^j,A^	0.67 ± 0.07 ^c,d,A^
V24	Onion (*A. cepa*) d.p.	91.40	0.69 ± 0.22 ^a,B^	16.70 ± 0.56 ^i,k,B^	0.095 ± 0.004 ^e,B^	0.36 ± 0.04 ^a,B^

All results have been expressed as the average value of three determination ± standard deviation. ^a–k^ A *t*-test was used to compare the mean differences registered for the macro elements among samples (V1–V24); data within the same column (V1–V24) sharing different superscripts are significantly different (*p* < 0.05). ^A–B^ highlights the significant differences between the i.p. and d.p. samples.

**Table 2:** Trace-element content in the 24 vegetable samples of different botanical families with permissible limits (EU and WHO/FAO) (μg·g^−1^ dry weight, mean ± SD).

Sample Coding	Vegetable Samples Analyzed	Cu	Mn	Fe	Cd	Pb	Zn	Co
Solanaceae								
V1	Tomato (*L. esculentum*) i.p.	32.5 ± 1.69 ^a,A^	13.00 ± 0.63 ^a,A^	33.50 ± 0.21 ^a,A^	0.10 ± 0.007 ^a^	0.75 ± 0.02 ^a,A^	4.50 ± 0.28 ^a,A^	N.D.
V2	Tomato (*L. esculentum*) d.p.	46.00 ± 4.10 ^b,B^	13.50 ± 0.28 ^a,A^	30.50 ± 0.28 ^a,A^	N.D.	0.65 ± 0.04 ^b,B^	9.50 ± 0.98 ^b,B^	2.20 ± 0.07 ^a^
V3	Bell Peppers (*C. annuum*) i.p.	60.00 ± 0.77 ^c,A^	27.50 ± 0.63 ^b,A^	62.50 ± 0.91 ^b,A^	0.10 ± 0.014 ^a,A^	0.75 ± 0.07 ^a,A^	17.50 ± 0.42 ^c,A^	0.85 ± 0.04 ^b,A^
V4	Bell Peppers (*C. annuum*) d.p.	30.50 ± 1.69 ^a,B^	22.50 ± 0.74 ^c,B^	60.50 ± 3.67 ^b,A^	0.50 ± 0.035 ^b,B^	0.80 ± 0.10 ^c,B^	20.50 ± 0.56 ^d,B^	3.20 ± 0.15 ^c,d,B^
V5	Eggplant (*S. melongena*) i.p.	76.50 ± 0.98 ^d,A^	28.50 ± 0.28 ^b,A^	51.00 ± 0.56 ^c,A^	0.15 ± 0.007 ^a^	0.75 ± 0.02 ^a,A^	11.00 ± 0.42 ^b,A^	N.D.
V6	Eggplant (*S. melongena*) d.p.	51.00 ± 1.90 ^e,B^	25.00 ± 0.98 ^b,c,A^	51.00 ± 0.77 ^c,A^	N.D.	0.50 ± 0.04 ^d,B^	16.00 ± 0.42 ^c,B^	2.90 ± 0.21 ^c^
V7	Potato (*S. tuberosum*) i.p.	8.00 ± 0.77 ^f,A^	12.50 ± 0.91 ^a,A^	62.50 ± 0.91 ^b,A^	N.D.	0.50 ± 0.05 ^d,A^	18.50 ± 0.91 ^d,A^	3.55 ± 0.17 ^d,A^
V8	Potato (*S. tuberosum*) d.p.	46.50 ± 3.67 ^b,B^	15.00 ± 0.91 ^a,d,A^	31.00 ± 0.77 ^a,B^	N.D.	0.70 ± 0.007 ^e,B^	5.50 ± 0.14 ^a,B^	2.90 ± 0.14 ^c,B^
Brassicaceae								
V9	Cauliflower (*B. oleracea* var. *botrytis*) i.p.	5.00 ± 0.63 ^f,A^	20.00 ± 1.13 ^c,e,A^	83.00 ± 2.68 ^d,A^	N.D.	0.20 ± 0.01 ^f,A^	28.50 ± 1.69 ^e,A^	0.50 ± 0.09 ^b,A^
V10	Cauliflower (*B. oleracea* var. *botrytis*) d.p.	5.00 ± 0.35 ^f,A^	16.00 ± 0.63 ^d,B^	110.00 ± 5.30 ^e,B^	N.D.	1.00 ± 0.1 ^g,B^	12.00 ± 1.20 ^b,B^	0.65 ± 0.03 ^b,A^
V11	White cabbage (*B. oleracea* var. *capitata*) i.p.	5.00 ± 0.56 ^f,A^	19.50 ± 0.91 ^b,A^	76.50 ± 3.53 ^f,A^	N.D.	0.65 ± 0.02 ^b,A^	9.00 ± 0.63 ^b,A^	3.15 ± 0.53 ^c,d^
V12	White cabbage (*B. oleracea* var. *capitata*) d.p.	6.00 ± 0.42 ^f,A^	17.50 ± 0.70 ^d,b,A^	72.00 ± 4.87 ^f,A^	0.25 ± 0.021 ^c^	0.80 ± 0.04 ^c,B^	4.50 ± 0.28 ^a,B^	N.D.
V13	Kohlrabie (*B. oleracea* var. *gongyloides*) i.p.	9.00 ± 0.77 ^f,A^	8.00 ± 0.35 ^f,A^	54.00 ± 2.68 ^c,A^	N.D.	0.60 ± 0.05 ^h,A^	8.00 ± 0.28 ^b,A^	N.D.
V14	Kohlrabie (*B. oleracea* var. *gongyloides*) d.p.	8.50 ± 0.49 ^f,A^	10.50 ± 1.13 ^a,f,A^	75.00 ± 0.63 ^f,B^	0.25 ± 0.014 ^c^	0.85 ± 0.04 ^i,B^	7.50 ± 0.28 ^b,A^	0.75 ± 0.09 ^b^
Apiaceae								
V15	Parsley (*P. crispum*) i.p.	14.00 ± 0.77 ^g,A^	29.50 ± 0.63 ^b,A^	94.00 ± 0.56 ^g,A^	N.D.	0.80 ± 0.04 ^c,A^	0.45 ± 0.10 ^a,A^	N.D.
V16	Parsley (*P. crispum*) d.p.	5.00 ± 0.63 ^f,B^	15.00 ± 0.49 ^d,B^	53.00 ± 1.41 ^c,B^	N.D.	0.75 ± 0.02 ^a,B^	0.90 ± 0.04 ^b,B^	2.15 ± 0.03 ^a^
V17	Carrot (*D. carota* subsp. *Sativus*) i.p.	0.50 ± 0.07 ^h,A^	11.50 ± 0.70 ^a,A^	78.50 ± 1.13 ^f,A^	0.30 ± 0.02 ^d^	0.85 ± 0.04 ^i^	0.85 ± 0.03 ^b,A^	N.D.
V18	Carrot (*D. carota* subsp. *Sativus*) d.p.	9.50 ± 0.98 ^f,B^	11.00 ± 0.77 ^a,A^	86.00 ± 0.63 ^d,B^	N.D.	N.D.	1.30 ± 0.14 ^f,B^	N.D.
V19	Celery (*A. graveolens*) i.p.	8.50 ± 0.63 ^f,A^	20.50 ± 0.84 ^b,A^	67.50 ± 1.83 ^b,A^	0.20 ± 0.007 ^c,A^	0.70 ± 0.01 ^e,A^	22.50 ± 1.41 ^d,A^	N.D.
V20	Celery (*A. graveolens*) d.p.	42.50 ± 1.69 ^b,B^	15.00 ± 0.14 ^d,B^	33.00 ± 0.28 ^a,B^	0.20 ± 0.014 ^c,A^	0.70 ± 0.04 ^e,A^	11.00 ± 0.56 ^b,B^	N.D.
Amaryllidaceae								
V21	Garlic (*A. sativum*) i.p.	10.00 ± 0.56 ^f,a,A^	10.00 ± 0.98 ^a,A^	55.00 ± 0.56 ^c,A^	N.D.	N.D.	1.85 ± 0.03 ^f,A^	N.D.
V22	Garlic (*A. sativum*) d.p.	5.50 ± 0.35 ^f,B^	10.00 ± 0.63 ^a,A^	38.00 ± 1.34 ^a,B^	0.40 ± 0.03 ^e^	0.80 ± 0.05 ^c^	1.70 ± 0.14 ^f,A^	N.D.
V23	Onion (*A. cepa*) i.p.	7.00 ± 0.63 ^f,A^	15.00 ± 1.34 ^d,A^	76.00 ± 1.27 ^f,A^	N.D.	N.D.	11.50 ± 0.63 ^b,A^	N.D.
V24	Onion (*A. cepa*) d.p.	31.50 ± 1.20 ^a,B^	11.50 ± 0.42 ^a,d,A^	18.50 ± 1.62 ^g,B^	0.25 ± 0.01 ^c^	0.75 ± 0.03 ^a^	3.50 ± 0.28 ^a,B^	N.D.
	WHO/FAO [40]	40	N.A.	450	0.2	0.3	60	N.A.
	EU [41]	20	500	N.A.	0.2	0.43	50	50

N.D.—Not detectable; N.A.—not applicable. All results have been expressed as the average value of three determination ± standard deviation. ^a–i^ A *t*-test was used to compare the mean differences registered for the trace elements among samples (V1–V24); data within the same column (V1–V24) sharing different superscripts are significantly different (*p* < 0.05). ^A–B^ highlights the significant differences between the i.p. and d.p. samples.

**Table 3:** Obtained results for the estimated daily intake (EDI) in the case of vegetable samples.

Sample Coding	EDI Cu (µg·Kg^−1^·day^−1^)	EDI Mn (µg·Kg^−1^·day^−1^)	EDI Fe (µg·Kg^−1^·day^−1^)	EDI Cd (µg·Kg^−1^·day^−1^)	EDI Pb (µg·Kg^−1^·day^−1^)	EDI Zn (µg·Kg^−1^·day^−1^)	EDI Co (µg·Kg^−1^·day^−1^)
V1	1.16 × 10^−3^	4.64 × 10^−4^	1.19 × 10^−3^	3.57 × 10^−6^	2.67 × 10^−5^	1.60 × 10^−4^	N.D.
V2	1.44 × 10^−3^	4.23 × 10^−4^	9.55 × 10^−4^	N.D.	2.04 × 10^−5^	2.97 × 10^−4^	6.89 × 10^−5^
V3	2.56 × 10^−3^	1.18 × 10^−3^	2.67 × 10^−3^	4.27 × 10^−6^	3.21 × 10^−5^	7.48 × 10^−4^	3.63 × 10^−5^
V4	1.23 × 10^−3^	9.08 × 10^−4^	2.44 × 10^−3^	2.02 × 10^−5^	3.23 × 10^−5^	8.27 × 10^−4^	1.29 × 10^−4^
V5	3.69 × 10^−3^	1.38 × 10^−3^	2.46 × 10^−3^	7.24 × 10^−6^	3.62 × 10^−5^	5.31 × 10^−4^	N.D.
V6	2.19 × 10^−3^	1.07 × 10^−3^	2.19 × 10^−3^	N.D.	2.14 × 10^−5^	6.86 × 10^−4^	1.24 × 10^−4^
V7	9.17 × 10^−4^	1.43 × 10^−3^	7.17 × 10^−3^	N.D.	5.73 × 10^−5^	2.12 × 10^−3^	4.07 × 10^−4^
V8	6.01 × 10^−3^	1.94 × 10^−3^	4.00 × 10^−3^	N.D.	9.04 × 10^−5^	7.10 × 10^−4^	3.75 × 10^−4^
V9	2.69 × 10^−4^	1.07 × 10^−3^	4.46 × 10^−3^	N.D.	1.07 × 10^−5^	1.53 × 10^−3^	2.69 × 10^−5^
V10	2.31 × 10^−4^	7.41 × 10^−4^	5.09 × 10^−3^	N.D.	4.63 × 10^−4^	5.55 × 10^−4^	3.01 × 10^−5^
V11	2.25 × 10^−4^	8.77 × 10^−4^	3.44 × 10^−3^	N.D.	2.92 × 10^−5^	4.05 × 10^−4^	1.42 × 10^−4^
V12	2.95 × 10^−4^	8.60 × 10^−4^	3.54 × 10^−3^	1.23 × 10^−5^	3.93 × 10^−5^	2.21 × 10^−4^	N.D.
V13	4.57 × 10^−4^	4.06 × 10^−4^	2.74 × 10^−3^	N.D.	3.04 × 10^−5^	4.06 × 10^−4^	N.D.
V14	4.60 × 10^−4^	5.68 × 10^−4^	4.06 × 10^−3^	1.35 × 10^−5^	4.60 × 10^−4^	4.06 × 10^−4^	4.06 × 10^−5^
V15	1.16 × 10^−3^	2.45 × 10^−3^	7.80 × 10^−3^	N.D.	6.64 × 10^−5^	3.74 × 10^−5^	N.D.
V16	3.94 × 10^−4^	1.18 × 10^−3^	4.18 × 10^−3^	N.D.	5.91 × 10^−5^	7.10 × 10^−5^	1.70 × 10^−4^
V17	2.89 × 10^−5^	6.64 × 10^−4^	4.54 × 10^−3^	1.73 × 10^−5^	4.91 × 10^−5^	4.91 × 10^−5^	N.D.
V18	5.61 × 10^−4^	6.50 × 10^−4^	5.08 × 10^−3^	N.D.	N.D.	7.68 × 10^−5^	N.D.
V19	2.33 × 10^−4^	5.61 × 10^−4^	1.85 × 10^−3^	5.47 × 10^−6^	1.92 × 10^−5^	6.16 × 10^−4^	N.D.
V20	1.14 × 10^−3^	4.02 × 10^−4^	8.84 × 10^−4^	5.36 × 10^−6^	1.88 × 10^−5^	2.95 × 10^−4^	N.D.
V21	2.18 × 10^−3^	2.18 × 10^−3^	1.20 × 10^−2^	N.D.	N.D.	4.04 × 10^−4^	N.D.
V22	1.15 × 10^−3^	2.09 × 10^−3^	7.94 × 10^−3^	8.35 × 10^−5^	1.67 × 10^−4^	3.55 × 10^−4^	N.D.
V23	3.40 × 10^−4^	7.29 × 10^−4^	3.69 × 10^−3^	N.D.	N.D.	5.59 × 10^−4^	N.D.
V24	1.55 × 10^−3^	5.65 × 10^−4^	9.09 × 10^−4^	1.23 × 10^−5^	3.69 × 10^−5^	1.72 × 10^−4^	N.D.

N.D.—Not detectable.

**Table 4:** Obtained results for the target hazard quotient (THQ), the total of the target hazard quotient (TTHQ) and the carcinogenic risk (CR) in the case of vegetable samples.

Sample Coding	THQ Cu	THQ Mn	THQ Fe	THQ Cd	THQ Pb	THQ Zn	THQ Co	TTHQ	CR Cd	CR Pb
V1	2.90 × 10^−2^	3.31 × 10^−3^	2.65 × 10^−5^	3.57 × 10^−3^	7.64 × 10^−3^	5.35 × 10^−4^	N.D.	4.41 × 10^−2^	4.99 × 10^−8^	3.74 × 10^−7^
V2	3.60 × 10^−2^	3.02 × 10^−3^	2.12 × 10^−5^	N.D.	5.82 × 10^−3^	9.92 × 10^−4^	6.89 × 10^−5^	4.93 × 10^−2^	N.D.	2.85 × 10^−7^
V3	6.41 × 10^−2^	8.40 × 10^−3^	5.94 × 10^−5^	4.27 × 10^−3^	9.16 × 10^−3^	2.49 × 10^−3^	3.63 × 10^−5^	9.03 × 10^−2^	5.98 × 10^−8^	4.49 × 10^−7^
V4	3.08 × 10^−2^	6.48 × 10^−3^	5.42 × 10^−5^	2.02 × 10^−2^	9.22 × 10^−3^	2.76 × 10^−3^	1.29 × 10^−4^	7.59 × 10^−2^	2.82 × 10^−7^	4.52 × 10^−7^
V5	9.23 × 10^−2^	9.83 × 10^−3^	5.47 × 10^−5^	7.24 × 10^−3^	1.03 × 10^−2^	1.77 × 10^−3^	N.D.	1.22 × 10^−1^	1.01 × 10^−7^	5.07 × 10^−7^
V6	5.46 × 10^−2^	7.65 × 10^−3^	4.86 × 10^−5^	N.D.	6.12 × 10^−3^	2.29 × 10^−3^	1.24 × 10^−4^	7.70 × 10^−2^	N.D.	3.00 × 10^−7^
V7	2.29 × 10^−2^	1.02 × 10^−2^	1.59 × 10^−4^	N.D.	1.64 × 10^−2^	7.07 × 10^−3^	4.07 × 10^−4^	7.71 × 10^−2^	N.D.	8.03 × 10^−7^
V8	1.50 × 10^−1^	1.38 × 10^−2^	8.90 × 10^−5^	N.D.	3.01 × 10^−2^	2.37 × 10^−3^	3.75 × 10^−4^	2.15 × 10^−1^	N.D.	1.27 × 10^−6^
V9	6.71 × 10^−3^	7.67 × 10^−3^	9.91 × 10^−5^	N.D.	3.07 × 10^−3^	5.10 × 10^−3^	2.69 × 10^−5^	2.40 × 10^−2^	N.D.	1.50 × 10^−7^
V10	5.79 × 10^−3^	5.29 × 10^−3^	1.13 × 10^−4^	N.D.	1.32 × 10^−2^	1.85 × 10^−3^	3.01 × 10^−5^	2.78 × 10^−2^	N.D.	6.48 × 10^−7^
V11	5.62 × 10^−3^	6.26 × 10^−3^	7.65 × 10^−5^	N.D.	8.35 × 10^−3^	1.35 × 10^−3^	1.42 × 10^−4^	2.87 × 10^−2^	N.D.	4.09 × 10^−7^
V12	7.37 × 10^−3^	6.14 × 10^−3^	7.86 × 10^−5^	1.23 × 10^−2^	1.12 × 10^−2^	7.37 × 10^−4^	N.D.	3.78 × 10^−2^	1.72 × 10^−7^	5.50 × 10^−7^
V13	1.14 × 10^−2^	2.90 × 10^−3^	6.09 × 10^−5^	N.D.	8.70 × 10^−3^	1.35 × 10^−3^	N.D.	2.44 × 10^−2^	N.D.	4.26 × 10^−7^
V14	1.15 × 10^−2^	4.06 × 10^−3^	9.02 × 10^−5^	1.35 × 10^−2^	1.31 × 10^−2^	1.35 × 10^−3^	4.06 × 10^−5^	4.57 × 10^−2^	1.89 × 10^−7^	6.44 × 10^−7^
V15	2.91 × 10^−2^	1.75 × 10^−2^	1.73 × 10^−4^	N.D.	1.90 × 10^−2^	1.25 × 10^−4^	N.D.	6.58 × 10^−2^	N.D.	9.30 × 10^−7^
V16	9.86 × 10^−3^	8.45 × 10^−3^	9.29 × 10^−5^	N.D.	1.69 × 10^−2^	2.37 × 10^−4^	1.70 × 10^−4^	4.40 × 10^−2^	N.D.	8.28 × 10^−7^
V17	7.22 × 10^−4^	4.75 × 10^−3^	1.01 × 10^−4^	1.73 × 10^−2^	1.40 × 10^−2^	1.64 × 10^−4^	N.D.	3.71 × 10^−2^	2.43 × 10^−7^	6.87 × 10^−7^
V18	1.40 × 10^−2^	4.64 × 10^−3^	1.13 × 10^−4^	N.D.	N.D.	2.56 × 10^−4^	N.D.	1.90 × 10^−2^	N.D.	N.D.
V19	5.82 × 10^−3^	4.01 × 10^−3^	4.11 × 10^−5^	5.47 × 10^−3^	5.47 × 10^−3^	2.05 × 10^−3^	N.D.	2.29 × 10^−2^	7.66 × 10^−8^	2.68 × 10^−7^
V20	2.85 × 10^−2^	2.87 × 10^−3^	1.97 × 10^−5^	5.36 × 10^−3^	5.36 × 10^−3^	9.83 × 10^−4^	N.D.	4.31 × 10^−2^	7.50 × 10^−8^	2.63 × 10^−7^
V21	5.45 × 10^−2^	1.56 × 10^−2^	2.67 × 10^−4^	N.D.	N.D.	1.35 × 10^−3^	N.D.	7.17 × 10^−2^	N.D.	N.D.
V22	2.87 × 10^−2^	1.49 × 10^−2^	1.76 × 10^−4^	8.35 × 10^−2^	4.77 × 10^−2^	1.18 × 10^−3^	N.D.	1.76 × 10^−1^	N.D.	2.34 × 10^−6^
V23	8.50 × 10^−3^	5.20 × 10^−3^	8.20 × 10^−5^	N.D.	N.D.	1.86 × 10^−3^	N.D.	1.56 × 10^−2^	N.D.	N.D.
V24	3.87 × 10^−2^	4.04 × 10^−3^	2.02 × 10^−5^	1.23 × 10^−2^	1.05 × 10^−2^	5.73 × 10^−4^	N.D.	6.61 × 10^−2^	N.D.	5.16 × 10^−7^

N.D.—Not detectable.

## Data Availability

All data is contained in the article.
